# Accurate prediction of asparagine deamidation in biologics using advanced machine learning models

**DOI:** 10.1093/bib/bbag363

**Published:** 2026-07-07

**Authors:** Shafayat Ahmed, Nicole Swope, Valentin Stanev, Romina Hofele, Dominique WuDunn, Rohan Jain, Jared Delmar, Maryam Pouryahya

**Affiliations:** Data Science and Modelling, Biopharmaceutical Development, AstraZeneca, 1 MedImmune Way, Gaithersburg, MD 20878, United States; Department of Computer Science, Virginia Tech, 620 Drillfield Dr., Blacksburg, VA 24060, United States; Analytical Sciences, Biopharmaceutical Development, AstraZeneca, 1 MedImmune Way, Gaithersburg, MD 20878, United States; Data Science and Modelling, Biopharmaceutical Development, AstraZeneca, 1 MedImmune Way, Gaithersburg, MD 20878, United States; Analytical Sciences, Biopharmaceutical Development, AstraZeneca, 1 MedImmune Way, Gaithersburg, MD 20878, United States; Analytical Sciences, Biopharmaceutical Development, AstraZeneca, 1 MedImmune Way, Gaithersburg, MD 20878, United States; Data Science and Modelling, Biopharmaceutical Development, AstraZeneca, 1 MedImmune Way, Gaithersburg, MD 20878, United States; Analytical Sciences, Biopharmaceutical Development, AstraZeneca, 1 MedImmune Way, Gaithersburg, MD 20878, United States; Data Science and Modelling, Biopharmaceutical Development, AstraZeneca, 1 MedImmune Way, Gaithersburg, MD 20878, United States

**Keywords:** GNN, PLM, ESM2, AlphaFold, fine-tuning, deamidation

## Abstract

The spontaneous deamidation of asparagine residues remains a major obstacle to the stability and efficacy of protein therapeutics. Currently available models in the literature for predicting deamidation liabilities can suffer from limited generalizability, likely due to biases such as sequence similarity within datasets. In this study, we built machine learning models using protein language models (PLMs, e.g. ESM2) and graph neural networks (GNNs), trained on a comprehensive dataset of 591 asparagine sites from over 105 protein molecules. To address the critical issue of data leakage, we implemented a peptide grouping strategy yielding more accurate estimates of model performance for novel deamidation sites. Our analysis shows that, when sequence similarity bias is controlled, PLMs match traditional feature-based models that use amino acid composition and predicted secondary structure/solvent accessibility, while offering substantial computational advantages. Additionally, our GNN-based pipeline further increases prediction accuracy by $\sim$8% compared with language model-only tools and delivers a 15%–25% improvement over motif-based approaches. This methodological framework enables more reliable and rapid *in-silico* prediction of deamidation liabilities, potentially reducing costly late-stage interventions in protein therapeutic development and is generalizable to the modeling of other protein post-translational modifications.

## Introduction

Protein therapeutics are a rapidly growing pharmaceutical segment, now accounting for about half of recent biologic drug approvals [1]. This class, which includes monoclonal antibodies, antibody–drug conjugates, fusion proteins, enzymes, and vaccines offers new treatment options for previously intractable diseases, but also faces unique development challenges, notably chemical degradation risks. Spontaneous deamidation of asparagine residues, forming aspartic or iso-aspartic acid, is among the most common, impacting drug activity, pharmacokinetics, and immunogenicity [2–4]. Accurate prediction and mitigation of asparagine deamidation is, therefore, critical for successful drug development.

Computational tools for deamidation prediction have evolved from sequence rule-based models that identify susceptible NG, NS, and NN motifs [5] to structure-informed and machine learning approaches that exploit predicted or homology-derived structural features [6–13]. Nevertheless, current models are limited, especially by inadequate handling of sequence similarity between training and test sets, which has frequently led to inflated performance estimates. This problem is particularly acute for antibody and antibody-derived therapeutics, where conserved or similarly engineered regions are common; random cross-validation can place related sequences in both training and validation/test partitions, resulting in data leakage and overstated metrics. Many prior models have been trained on commercially available or in-house biologics that exhibit substantial within-dataset sequence similarity, further exacerbating these issues.

Recent advances, most notably AlphaFold2 for structure prediction [14] and protein language models (PLMs) such as ESM2 for deamidation prediction [15], offer promising avenues to mitigate these limitations. AlphaFold2 enables structural modeling in the absence of suitable templates, while PLMs leverage large-scale sequence corpora to infer functional properties without explicit structural inputs, potentially enhancing generalizability, robustness, and computational efficiency.

We developed an improved framework for deamidation prediction by curating a large unbiased dataset and preventing data leakage, showing that PLMs offer reliable, scalable assessment for protein therapeutics. To summarize our main contributions:

- Rigorous Data Integrity Assessment: We thoroughly investigated the influence of data splitting strategies on prediction validity, demonstrating that grouping sequence-similar peptides in the same fold is essential for preventing data leakage and yielding accurate generalization metrics. We also quantified how different sequence window sizes surrounding Asn sites affect model sensitivity and specificity, supporting rational parameter selection.
- Benchmarking of Prediction Pipelines: We evaluated four prediction pipelines spanning conventional sequence- and structure-derived features, PLM embeddings, and graph-based structural representations. This analysis compared alternative feature sets, PLM variants, and graph neural network (GNN) architectures to determine which combinations were most effective for deamidation prediction.
- Practical Implications: Our work establishes a robust computational framework capable of rapid, scalable, and reliable *in-silico* deamidation liability assessment. This significantly reduces the risk of late-stage pipeline failure due to chemical instability and enables its integration into early phase screening of biopharmaceutical candidates.

## Experiments and data collection

### Sample preparation and LC-MS/MS analysis

Therapeutic protein samples at 10 mg/mL were incubated at 40$^{\circ }$ and pH 6.0 for $\sim$4 weeks to accelerate deamidation. This stress condition was selected as more representative of manufacturing and storage conditions compared with the pH 8.0 conditions used in our previous study [16]. Samples were collected at 0-, 2-, and 4-week timepoints and stored at $-80^{\circ }$ prior to analysis.

LC-MS/MS tryptic peptide mapping was performed following the protocol described in our previous work [16], with modifications for improved site localization. Briefly, samples were denatured, reduced, alkylated, and digested with trypsin before separation on a reversed-phase C18 column and analysis by high-resolution mass spectrometry. Deamidation quantitation was based on extracted ion chromatography peak areas, with site-specific assignments confirmed by MS/MS fragmentation when possible.

### Protein structure generation and feature extraction

Full-length protein structures were generated using AlphaFold2 Multimer (v2.3.2) [17], representing a significant advancement over the homology modeling approach used in our previous work [11, 16]. This method enables accurate structure prediction for molecules lacking suitable homologous templates in the Protein Data Bank, particularly for non-antibody proteins. For monoclonal antibodies, structures were truncated to the variable fragment (Fv) region to prevent the appearance of redundant conserved asparagine sites in our training dataset, while retaining all valuable variable region asparagine sites relevant to deamidation prediction.

For the structure-based model, features were extracted for each asparagine residue using custom Python scripts utilizing mainly the Biopython package (v1.80), following a similar framework established in our previous study, but significantly augmented to include additional valuable features. These parameters included: (i) N+1 residue identity as both a categorical variable and empirical pentapeptide half-life (pphl) [18], (ii) backbone dihedral angles (phi and psi) of N, N+1, and N-1 residues, (iii) asparagine side chain dihedrals (chi1 and chi2), (iv) nucleophilic attack distance between the side chain carbonyl and backbone nitrogen, (v) solvent accessibility expressed as both percentage and absolute area, (vi) hydrogen bonding to the asparagine side chain, (vii) secondary structure classification of N, N+1, and N-1 residues, (viii) IMGT properties of N+1 and N-1 residues, including volume, hydrophobic moment, isoelectric point, polarity, and charge [19], and (ix) local formal charge in the vicinity of the backbone N+1 residue nitrogen. These parameters captured the relevant local structural environment and neighboring residue properties as described previously [11].

### Data quality control and preprocessing

Rigorous data cleaning procedures were implemented to ensure training set quality. Asparagine sites with ambiguous mass spectrometric assignments were excluded from the dataset. This included cases where multiple asparagines occurred within a single tryptic peptide without sufficient MS/MS fragmentation data to localize the modification site. Additionally, sites from peptides with known complex deamidation patterns (such as the conserved “PENNY” motif [20]) were handled based on extensive experience with this conserved peptide in order to quantify individual site contributions from peptide-level measurements. Asparagine residues followed by proline (“NP sites” where N+1=P) were removed from the dataset, which is a preprocessing step not seen in the literature or our previous publication. This is important because NP sites cannot undergo deamidation; the absence of a backbone amid NH on proline prevents nucleophilic attack on the Asn side chain carbonyl carbon atom and subsequent succinimide ring formation. As all NP sites will belong to the negative deamidation class, and the backbone structure is significantly different and unique from all other sites, removal of these sites improves learning of the model on relevant sites and structures [12].

**Figure 1.**
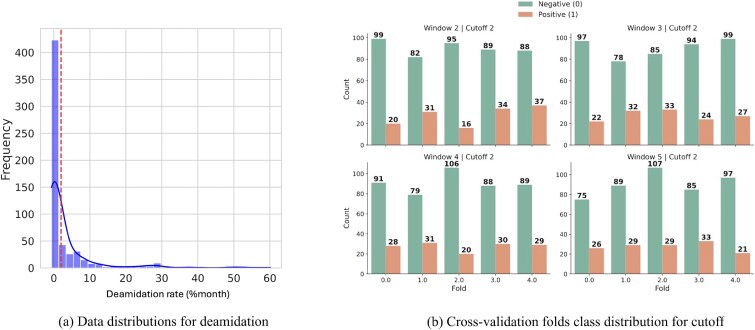
Data distribution of the collected samples and their deamidation rates. (a) Problematic antibodies are characterized by higher values further from zero, as indicated by the red dashed line. (b) Count of positive and negative samples per fold for deamidation cutoff rate 5, illustrating the sample balance across windows and experimental groups.

Deamidation rates were calculated by least-squares fitting to an exponential decay function, and sites were classified as deamidating (TRUE) or non-deamidating (FALSE) based on a threshold of both $\geq$2.0% and $\geq$5.0% deamidation per month under stress conditions. 5.0% is a meaningful threshold for antibodies, in particular, as deamidation in critical regions for activity (CDRs) could result in a 10% loss in activity if binding of both Fab arms are required. Here 2.0% is approaching the error of the experimental method used, resulting in a model that identifies all deamidation sites regardless of severity. In total, the dataset comprises 591 sites, of which 485 are negative (non-deamidating) and 106 are positive (deamidating) according to the 5.0% cutoff threshold (see Fig. 1).

## Methodology

### Problem formulation

We formulate deamidation prediction as a binary classification problem. Each sample corresponds to an asparagine residue and is labeled as positive if its experimentally measured deamidation rate exceeds a predefined cutoff ($\geq$2.0% or $\geq$5.0%), and negative otherwise. The 5.0% threshold was chosen as a functionally relevant cutoff for antibodies, since deamidation in critical regions such as CDRs can impair activity and, when binding requires both Fab arms, may correspond to an $\sim$10% loss of function. The 2.0% threshold was included as a more permissive cutoff because it approaches the experimental error of the assay, allowing detection of deamidation-prone sites regardless of severity.

### Data splitting strategies

Careful data splitting is essential for fair evaluation of predictive models. If information from the test set leaks into training, or if highly similar samples appear in both sets, performance may be inflated. Conversely, suboptimal splitting can underestimate accuracy when splits are unbalanced or unrepresentative.

#### Peptide group data splitting

Because asparagine deamidation is primarily determined by local sequence context rather than whole-protein similarity, we define groups using peptide windows centered on the target asparagine rather than full-sequence clustering. The grouping strategy is designed specifically to control leakage of local flanking motifs between training and test folds. We defined a window size to specify the local sequence context used to group residues for the train–test split. Each window is centered on the target asparagine (N) and grows outward by alternately extending to the N-terminus and C-terminus of the sequence. For example, size 1 includes [N, N+1], size 2 expands to [N-1, N, N+1], size 3 to [N-1, N, N+1, N+2], and size 4 to [N-2, N-1, N, N+1, N+2], and so on. If multiple residues have the same sequence within their defined window, they are grouped together into clusters. Each cluster is used as the basic grouping for assigning samples to cross-validation folds, preventing highly similar local sequence contexts from appearing in both the training and test sets.

**Figure 2.**
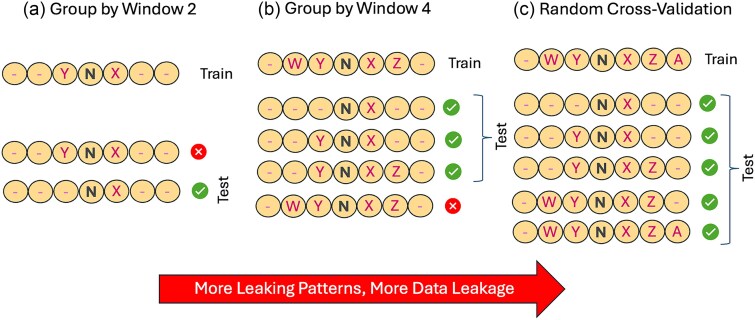
Effect of grouping window on data leakage. In the absence of grouping (C: random cross-validation), overlapping sequence contexts freely occur in both training and test sets, leading to substantial leakage and inflated performance estimates. Grouping reduces leakage by keeping similar windows within the same split; however, as the window size increases, more contexts merge, and residual leakage rises. In the limit of very large windows, grouping converges toward random cross-validation because nearly all contexts overlap.

Window size directly influences the risk of information leakage between training and test sets in our clustering approach. Smaller windows, which represent short sequence motifs around the asparagine, are more likely to recur across different peptides, resulting in larger clusters that group together many residues sharing the same local context. By assigning each cluster wholly to either the training or test set, we ensure that identical local motifs do not span both splits and thus strictly minimize data leakage. Conversely, larger windows capture extended and specific sequence contexts, leading to smaller clusters with less recurrence. In this scenario, key local motifs may be distributed across different clusters and assigned to both training and test sets, increasing the possibility for information leakage through repeated sequence features. By varying the window size, we can modulate the stringency of this grouping strategy. Small windows produce highly conservative partitions with minimal leakage, while large windows relax this constraint and potentially allow more overlap, enabling robust evaluation of model generalization and sensitivity to local context (Fig. 2).

For each window size and deamidation cutoff, splits are created using StratifiedGroupKFold algorithm [21] which balances both the number of samples and the positive/negative class ratio as much as possible. Additional iterative swaps further refine class balance within a tolerance (see Fig. 1b).

**Figure 3.**
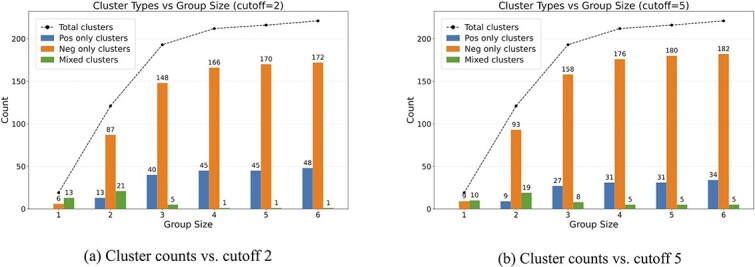
Positive, negative, and mixed cluster counts for grouping sizes 1–6 at cutoffs 2 and 5. Mixed clusters level off by window size 5, indicating a plateau in data leakage. Beyond window 5, no new overlapping patterns appear between train and test.

Across window sizes 1–6 and both cutoffs (2.0% and 5.0%), the number of unique clusters increases with window size and then saturates (dashed line in Fig. 3; detailed per-cutoff views in Fig 3) . In all settings, most clusters are negative-only (orange), consistent with the strong class imbalance (few deamidated residues). Mixed clusters (containing both positive and negative samples) decline rapidly as window size grows and reach a stable plateau by window size 4 for both cutoffs. This suggests that a four-residue flanking window is already near-sufficient to separate deamidated from non-deamidated residues; enlarging the window beyond this point adds redundant contextual information without introducing new discriminative patterns (Fig. 2). Consequently, the risk of data leakage also plateaus around window size 5.

#### Nested cross-validation

To rigorously assess model performance on data that differ from the training distribution, such as sequences or molecular graphs with novel motifs, domains, or feature combinations not seen during training, we employed a nested cross-validation framework for both the PLM and GNN pipelines. For each grouping strategy and cutoff setting, the dataset was first partitioned into five fixed stratified group folds. In the outer loop, five runs were performed, where in each run one fold was held out as the external test set and the remaining four folds were used as the training pool. This process was repeated until each fold served as the test set. Model performance was then summarized as the mean and SD across the five outer test folds.

Within each outer-loop run, hyperparameter selection was performed using an inner four-fold cross-validation procedure on the four-fold training pool. In each inner iteration, three folds were used for training and one fold was used for validation, rotating across all four-folds. Hyperparameters were selected based on the mean validation F1-score across the inner folds, as F1-score provides a balanced criterion under class imbalance. After selecting the best hyperparameter configuration, the final model was retrained on all four outer training folds and evaluated once on the held-out outer test fold. This design ensured strict separation between model selection and final testing, thereby providing an unbiased estimate of generalization performance.

### Overview of prediction approaches

#### Motif-based approach

As noted earlier, we use a motif-driven approach grounded in known biochemical patterns of spontaneous asparagine (N) deamidation [5]. In this sequence-level heuristic, a residue is labeled deamidation-prone when an asparagine is immediately followed by glycine (G), serine (S), or asparagine (N). The method is structure-independent, fast, and interpretable, serving as a clear baseline for comparison. Accordingly, it has consistently been included in our results tables for evaluation.

#### Feature-based models

A machine learning classifier was used to predict developability properties from selected protein features, as detailed in Section “Protein structure generation and feature extraction.”

The molecular descriptors included both sequence-based features, such as pphl values and IMGT properties of the N+1 and N–1 residues, and structure-based features, specifically backbone dihedral angles (phi and psi) for the N, N+1, and N–1 residues, as well as asparagine side chain dihedrals (chi1 and chi2), nucleophilic attack distance, solvent accessibility, hydrogen bonding, secondary structure, and local charge. The performance of models using only sequence-based features was compared with those incorporating both sequence- and structure-based features. Notably, sequence-derived features can be computed directly from the protein sequence, obviating the need for structure prediction and substantially reducing computational cost.

To further assess the impact of structural variability and data balance, we experimented with including either the top-ranked AlphaFold2 predicted structure or augmenting the minority class by adding more structural models. Specifically, during training, we balanced the dataset by incorporating the Top25 most probable AlphaFold2-predicted structures for the minority class. Model validation was performed on the same fold split of the validation set without any structural augmentation, allowing for a robust and fair comparison to models relying only on the top-ranked structure.

#### Protein language model

We utilize a state-of-the-art PLM, ESM-2, which is an evolutionary-scale transformer architecture pretrained on large-scale protein sequence databases [22]. This model captures rich sequence context and biochemical properties across diverse protein families, making it well-suited for protein modeling tasks. To adapt ESM-2 representations to our deamidation prediction task, we explore and systematically compare three distinct training regimes:

- **Frozen PLM:** In this configuration, all transformer weights of the ESM-2 backbone are kept fixed throughout training. Only the downstream feed-forward multilayer perceptron (MLP) classifier head responsible for mapping the PLM’s contextual representations to prediction outputs is trained on the labeled therapeutic protein data. By leveraging solely the pretrained sequence features without additional adaptation, this approach minimizes the risk of overfitting (particularly valuable in limited-data scenarios) while retaining all general protein knowledge learned during pretraining.
- **Full fine-tuning:** Here, both the ESM-2 backbone and the MLP head are jointly optimized end-to-end with respect to the deamidation prediction loss function. All self-attention layers, feed-forward blocks, and layer normalization statistics in the transformer are allowed to update during training. As a result, the model can fully adapt its sequence context encoding to the therapeutic protein-specific domain and the nuances of deamidation rate, potentially learning task-adapted representations. However, this regime greatly increases the number of trainable parameters and may be prone to overfitting in low-data regimes unless regularization is employed.
- **LoRA fine-tuning:** We incorporate Low-Rank Adaptation (LoRA), a parameter-efficient training strategy, into selected attention layers of ESM-2. Instead of updating the full transformer, LoRA inserts low-rank learnable adapters into the query, key, and value matrix projections (and optionally in output or intermediate dense layers). Only a small subset of newly introduced low-rank weights is trained, while the majority of the pretrained model remains frozen. This approach enables targeted, efficient model adaptation while notably reducing computational burden and mitigating the risk of overfitting relative to full-parameter fine-tuning. LoRA layers provide the model with extra capacity to adjust to therapeutic protein specific context without sacrificing inference speed or memory.

In all regimes, the transformer outputs serve as input features to a downstream MLP classifier head. The MLP receives contextualized residue representations from ESM-2 and predicts the probability of deamidation for specific sequence positions.

A key aspect of our approach is the use of variable-size windows around each target residue. For residue $i$, the model considers either a single position or a symmetric window comprising $w$ residues (with $w$ treated as a tunable hyperparameter) centered on $i$. The embeddings for these windowed residues are either concatenated or averaged (mean pooled), depending on the optimization setting. Only these fixed window residues are used for each classification decision, ensuring the model leverages both local sequence context and the rich representations derived from the PLM.

The MLP head itself comprises a hidden layer with ReLU activation, an optional dropout for regularization, and a final output layer mapping to the two deamidation classes. Throughout training, the window size $w$ is varied systematically to identify the optimal local context for deamidation prediction. This flexible windowed approach allows us to assess the importance of neighboring residues, balancing local information with long-range PLM-derived sequence features.

All model variants and hyperparameters are benchmarked to identify the most effective strategy for capturing therapeutic protein deamidation patterns.

#### Graph neural network

**Figure 4.**
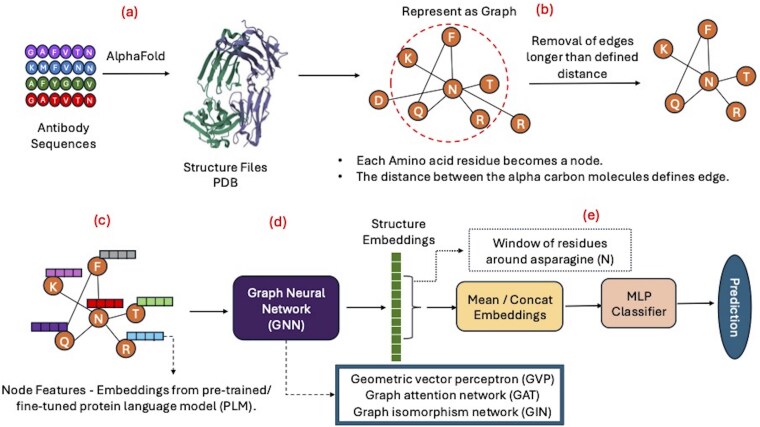
Overview of the GNN architecture for deamidation prediction. (a) Therapeutic protein sequences are first folded via AlphaFold to obtain 3D structural models. (b) The resulting structures are converted into residue-level graphs, where each node represents an amino acid and edges connect residues based on defined spatial distance thresholds. (c) Node features are computed using sequence embeddings generated by a pretrained or a fine-tuned PLM, capturing local biochemical context. (d) These graphs and node features are then processed by a GNN model such as geometric vector perceptron (GVP), graph attention network (GAT), or graph isomorphism network (GIN) to produce structure-aware residue embeddings. (e) For each specified asparagine site targeted for deamidation prediction, a fixed window of neighboring residues is extracted. The embeddings for residues within this window are aggregated and passed through a multi-layer perceptron (MLP) classifier to assess deamidation risk.

To leverage structural context, each protein structure predicted by AlphaFold is converted into a residue-level graph (see Fig. 4). In this graph, nodes correspond to residues, initialized with deep contextual features, and edges encode geometric relationships between spatially proximal residues. Our framework systematically explores architectural and training hyperparameters to optimize predictive performance.

- **GNN Backbone:** We evaluate three GNN architectures as the learnable backbone: GVP, GAT, and GIN. Node embeddings are initialized using PLM outputs and are propagated through multiple rounds of message passing, where the depth (number of layers) is a hyperparameter.
- **Graph Construction:** For every therapeutic protein, a residue graph is constructed as follows:
  \begin{align*} \textrm{edge}(i, j) \iff \|\mathbf{x}_{i}^{C_\alpha} - \mathbf{x}_{j}^{C_\alpha}\|_{2} \leq r \end{align*}
  Edges are assigned when the $C_\alpha$ atoms of residues $i$ and $j$ are within a spatial cutoff $r$. Connectivity is further restricted to the $k$ nearest spatial neighbors, making both $r$ and $k$ tunable hyperparameters.
- **Node and Edge Features:**
 
  \begin{align*} \mathbf{h}_{i} &= \textrm{PLM}(\textrm{seq})[i] \\ d_{ij} &= \|\mathbf{x}_{j}^{C_\alpha} - \mathbf{x}_{i}^{C_\alpha}\|_{2} \\ \mathbf{v}_{ij} &= \frac{\mathbf{x}_{j}^{C_\alpha} - \mathbf{x}_{i}^{C_\alpha}}{\|\mathbf{x}_{j}^{C_\alpha} - \mathbf{x}_{i}^{C_\alpha}\|_{2}} \\ \mathbf{e}_{ij} &= (d_{ij}, \mathbf{v}_{ij}) \end{align*}
  Node features encode PLM-derived embeddings; edge features encode geometric proximity and spatial orientation.
- **Neighborhood Selection:** Edges are included only if both distance and $k$-nearest neighbor criteria are met, controlling graph density and biological relevance through hyperparameters $r$ and $k$.
- **Windowed Prediction:** After $L$ message passing layers, node embeddings are pooled over a fixed-size window $w$ centered at each target asparagine residue. The window size $w$ and pooling method (concatenation or mean) are systematically varied. Only windowed residues are included for each prediction:
  \begin{align*} & (\mathbf{h}_{i-w/2}, \ldots, \mathbf{h}_{i+w/2}) \rightarrow \textrm{MLP} \end{align*}
  The pooled window embedding is passed to an MLP that predicts deamidation status for the center residue.
- **Hyperparameters and Training Criteria:** The following hyperparameters are optimized through cross-validation:
  – **GNN backbone**: GVP, GAT, or GIN.
  – **Number of GNN layers** ($L$).
  – **Spatial cutoff distance** ($r$): $10$, $15$, or $20$ Å.
  – **Neighborhood size** ($k$): $10$, $20$, or $30$ neighbors per node.
  – **MLP window size** ($w$) and **pooling strategy**: window sizes $1$–$5$ with mean or concatenation pooling.
  – **Loss function**: cross-entropy or focal loss (parameter $\gamma$), where focal loss down-weights well-classified examples to address class imbalance.
  – **Early stopping**: based on validation loss to prevent overfitting and enhance generalization.
- **Evaluation:** All models were trained end-to-end using nested cross-validation to rigorously assess generalization to out-of-distribution therapeutic proteins. Here, “out-of-distribution” refers to test sequences that differ from the training distribution in sequence identity and motif/context composition, achieved by group-aware splits that prevent closely related sequences from appearing in both train and test folds. We report classification accuracy, precision, recall, F1-score, ROC–AUC, and area under the precision–recall curve (AUPRC).

## Results and comparison

### Performance of motif- and feature-based models

Table 1 reports the performance of rule- and feature-based methods for predicting Asparagine (N) deamidation sites. The motif-based baseline, which relies exclusively on next-residue deamidation-site motifs, achieved moderate accuracy (0.63) and the highest sensitivity (0.63) across models, but its F1-score (0.40) lagged behind feature-driven approaches. A model leveraging sequence-derived features attained higher accuracy (0.76) and AUC (0.66) but exhibited lower F1-score (0.33) and recall (0.37). Introducing structural features markedly improved performance. The Top1 Structure model achieved higher accuracy (0.83) and the highest F1-score (0.49), indicating the most favorable precision–recall trade-off among all methods. The Top25 Augmented model, which enhances minority-class representation through the Top25 predicted structural augmentations, delivered the highest AUPRC and a slightly higher F1-score than Top1 Structure, but with greater variance, and did not surpass Top1 Structure on other metrics. Of note, the augmented approach incurs substantially higher computational cost to compute features across the Top25 structures, and thus would require further performance gains to justify this overhead.

**Table 1:** Comparison of cross-validation performance (mean ± SD) for feature-based models on G4_C5 (where G denotes the grouping scheme and C the deamidation cutoffs), alongside a motif-based baseline that uses sequence patterns only, while feature-based models incorporate sequence and structural descriptors, with structure augmentation applied to the minority class.

Model	Accuracy	Precision	Recall	F1-Score	ROC–AUC	AUPRC
Motif-based	0.63 $\pm$ 0.15	0.30 $\pm$ 0.18	**0.63** $\pm$ 0.24	0.40 $\pm$ 0.20	0.63 $\pm$ 0.18	0.29 $\pm$ 0.15
Sequence-based	0.76 $\pm$ 0.15	0.32 $\pm$ 0.25	0.37 $\pm$ 0.24	0.33 $\pm$ 0.15	0.66 $\pm$ 0.22	0.33 $\pm$ 0.19
Top1-structure	**0.83** $\pm$ 0.05	**0.54** $\pm$ 0.16	0.50 $\pm$ 0.26	**0.49** $\pm$ 0.16	**0.70** $\pm$ 0.11	0.59 $\pm$ 0.22
Top25-augmented	**0.83** $\pm$ 0.15	0.48 $\pm$ 0.19	0.58 $\pm$ 0.24	**0.51** $\pm$ 0.25	0.65 $\pm$ 0.23	**0.68** $\pm$ 0.25

The highest value for each metric is both bolded and underlined for clarity.

In summary, Top1 Structure offers the most balanced performance, optimizing both accuracy and F1-score, and is therefore selected for subsequent comparisons against PLM- and GNN-based models.

### Performance of protein language model and graph neural network models

Table 2 reports cross-validation performance for PLM and GNN models, benchmarked against Motif-based and Top1-structure baselines, across five independent group-cutoff-5 splits. Similar to feature-based models, PLM and GNN configurations were selected to maximize F1-score, providing a robust criterion under class imbalance and precision–recall trade-offs.

**Table 2:** Cross-validation performance (mean ± SD) for GNN, PLM, Motif, and Top1-Structure models across different group–cutoff splits, where Gx_Cy denotes the split configuration with G specifying the grouping scheme used to prevent leakage and C the deamidation rate cutoff used to separate good and bad molecules.

Data	Model	Accuracy	Precision	Recall	F1-Score	ROC-AUC	AUPRC
G1_C5	GNN	0.70 $\pm$ 0.19	0.38 $\pm$ 0.08	0.76 $\pm$ 0.27	0.48 $\pm$ 0.11	0.69 $\pm$ 0.19	0.39 $\pm$ 0.17
	PLM	0.47 $\pm$ 0.13	0.23 $\pm$ 0.08	**0.93** $\pm$ 0.15	0.36 $\pm$ 0.11	0.61 $\pm$ 0.20	0.32 $\pm$ 0.21
	Motif-based	0.65 $\pm$ 0.26	0.17 $\pm$ 0.22	0.57 $\pm$ 0.52	0.25 $\pm$ 0.28	0.63 $\pm$ 0.16	0.25 $\pm$ 0.16
	Top1-structure	**0.80** $\pm$ 0.08	**0.42** $\pm$ 0.20	0.68 $\pm$ 0.31	**0.49** $\pm$ 0.22	**0.76** $\pm$ 0.14	**0.47** $\pm$0.19
G2_C5	GNN	**0.77** $\pm$ 0.09	0.39 $\pm$ 0.13	0.66 $\pm$ 0.29	**0.48** $\pm$ 0.19	**0.78** $\pm$ 0.10	**0.48** $\pm$ 0.19
	PLM	0.69 $\pm$ 0.24	**0.41** $\pm$ 0.25	0.63 $\pm$ 0.25	0.45 $\pm$ 0.23	0.70 $\pm$ 0.19	0.38 $\pm$ 0.23
	Motif-based	0.63 $\pm$ 0.17	0.27 $\pm$ 0.16	**0.68** $\pm$ 0.38	0.38 $\pm$ 0.22	0.66 $\pm$ 0.18	0.29 $\pm$ 0.08
	Top1-structure	0.76 $\pm$ 0.09	0.36 $\pm$ 0.07	0.40 $\pm$ 0.20	0.35 $\pm$ 0.10	0.62 $\pm$ 0.06	0.35 $\pm$ 0.08
G3_C5	GNN	0.85 $\pm$ 0.05	0.56 $\pm$ 0.17	0.66 $\pm$ 0.25	0.59 $\pm$ 0.17	**0.81** $\pm$ 0.13	0.57 $\pm$ 0.22
	PLM	**0.86** $\pm$ 0.06	**0.67** $\pm$ 0.21	0.49 $\pm$ 0.23	0.54 $\pm$ 0.18	0.75 $\pm$ 0.17	**0.58** $\pm$ 0.22
	Motif-based	0.64 $\pm$ 0.09	0.27 $\pm$ 0.13	0.64 $\pm$ 0.30	0.37 $\pm$ 0.17	0.64 $\pm$ 0.13	0.26 $\pm$ 0.11
	Top1-structure	0.79 $\pm$ 0.10	0.49 $\pm$ 0.15	**0.83** $\pm$ 0.09	**0.60** $\pm$ 0.10	0.80 $\pm$ 0.05	0.52 $\pm$ 0.13
G4_C5	GNN	0.81 $\pm$ 0.12	**0.60** $\pm$ 0.23	0.62 $\pm$ 0.18	**0.57** $\pm$ 0.13	0.77 $\pm$ 0.16	0.52 $\pm$ 0.17
	PLM	0.80 $\pm$ 0.07	0.50 $\pm$ 0.09	**0.70** $\pm$ 0.23	0.56 $\pm$ 0.11	**0.83** $\pm$ 0.10	**0.62** $\pm$ 0.21
	Motif-based	0.63 $\pm$ 0.15	0.30 $\pm$ 0.18	0.63 $\pm$ 0.24	0.40 $\pm$ 0.20	0.63 $\pm$ 0.18	0.29 $\pm$ 0.15
	Top1-structure	**0.83** $\pm$ 0.05	0.54 $\pm$ 0.16	0.50 $\pm$ 0.26	0.49 $\pm$ 0.16	0.70 $\pm$ 0.11	0.59 $\pm$ 0.22
G5_C5	GNN	0.83 $\pm$ 0.06	0.55 $\pm$ 0.11	**0.85** $\pm$ 0.18	**0.65** $\pm$ 0.09	**0.91** $\pm$ 0.06	**0.74** $\pm$ 0.13
	PLM	0.82 $\pm$ 0.18	**0.71** $\pm$ 0.30	0.69 $\pm$ 0.16	**0.65** $\pm$ 0.23	0.85 $\pm$ 0.13	0.69 $\pm$ 0.23
	Motif-based	0.64 $\pm$ 0.11	0.29 $\pm$ 0.17	0.64 $\pm$ 0.31	0.38 $\pm$ 0.20	0.64 $\pm$ 0.18	0.28 $\pm$ 0.11
	Top1-structure	**0.84** $\pm$ 0.05	0.54 $\pm$ 0.11	0.58 $\pm$ 0.21	0.55 $\pm$ 0.15	0.74 $\pm$ 0.11	0.60 $\pm$ 0.23

For each split, the highest mean value within a metric column is shown in bold and underlined for clarity.

Performance trends were consistent across cutoff settings. Results for group-cutoff-2 were similar to those for group-cutoff-5, suggesting the models are not overly sensitive to the cutoff stringency. We present the cutoff-5 results in the main text to reflect a more conservative generalization assessment, while the corresponding cutoff-2 results are provided in Supplementary Table S1 for completeness. Under the F1-driven tuning, GNN consistently attains the highest mean F1 in three splits (G2_C5, G4_C5, and G5_C5) and produces F1 values effectively equivalent to the top performer in the remaining two splits (G1_C5, G3_C5).

PLM models display a complementary profile: they achieve high precision in several splits (notably G3_C5 and G5_C5) and obtain the highest mean accuracy in G3_C5. These results indicate that the PLM configurations tuned for F1 tend to reduce false positives, often at the expense of recall relative to GNN.

Top1-structure models attain the single highest mean F1 in G1_C5 and show consistently high accuracy across multiple splits, but they do not consistently outperform GNN on F1. In some splits (e.g. G3_C5) Top1-structure exhibits higher recall than other models; however, elevated accuracy does not translate into a systematic F1 advantage, underscoring the well-known limitations of accuracy as a primary metric on imbalanced problems such as deamidation site prediction.

The relatively large standard deviations observed for some settings, particularly under stricter group-aware splits such as G1_C5, reflect substantial fold-to-fold variability caused primarily by class imbalance combined with group-constrained data partitioning. Although folds were constructed to be as stratified as possible, the requirement that each peptide-window cluster remain entirely within a single fold limits how evenly positive samples can be distributed. Consequently, some outer test folds contain only a small number of positive sites, making metrics such as recall especially sensitive to small differences in the number of correctly recovered positives. This effect is most pronounced for simpler baselines such as the motif-based model and should be interpreted in the context of limited positive support per fold rather than model instability alone. To reiterate, although the class imbalance and resulting fold-to-fold variability is undesirable, it is a byproduct of our group-constrained data splitting strategy and necessary for a realistic performance measure on never-seen deamidation motifs.

Motif-based models underperform relative to the other approaches across nearly all metrics and splits. Mean values for accuracy, ROC–AUC, AUPRC, precision, recall, and F1 are consistently lower for motif-only predictors, indicating that motif information alone provides limited discriminatory power in this dataset.

Overall, GNN achieves the best average F1 (0.55), leading or on par on G2_C5 (0.48), G4_C5 (0.57), and G5_C5 (0.65). Top1-structure achieved the highest F1-score on the G1_C5, narrowly exceeding GNN on F1 in G1_C5 (0.49 versus 0.48) and G3_C5 (0.60 versus 0.59). With respect to accuracy, Top1-structure is frequently highest (G1_C5: 0.80; G4_C5: 0.83; G5_C5: 0.84), while GNN leads in G2_C5 and PLM leads in G3_C5. On ranking metrics, GNN often attains top ROC–AUC and AUPRC values, with Top1-structure leading in G1_C5 and PLM in G4_C5.

Trends observed across grouping windows (G1–G5) and cutoffs (C2 and C5) are broadly consistent (Supplementary Fig. S1). Across these additional analyses, GNN typically achieves higher recall, F1, and accuracy, particularly on the more challenging splits (G1–G3). At cutoff-2, GNN outperforms the PLM pipeline in nearly all groups except Group 3, where PLM leads marginally; at cutoff-5, PLM attains modestly higher recall in Groups 1, 4, and 6 but with reduced precision (Supplementary Fig. S1). Collectively, these results indicate that the structure-aware methods outperform the sequence-only baseline, with GNN delivering the most consistent F1 and ranking improvements across C5 and Top1-structure yielding the highest accuracy on several difficult splits.

**Figure 5.**
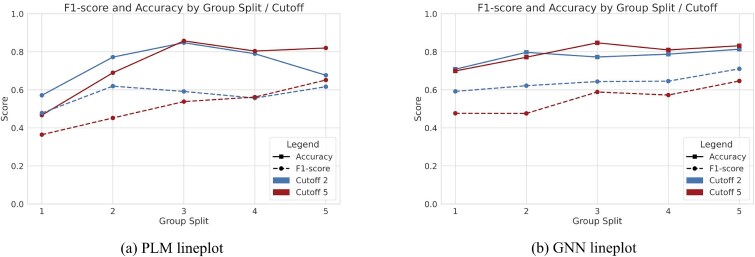
Accuracy and F1-score comparison across group splits (G1–G5) and cutoffs (C2, C5) for both (a) PLM and (b) GNN pipelines.

Figure 5 shows F1-score and accuracy across group splits (G1–G5) and cutoffs (C2, C5) for both PLM and GNN pipelines. Performance improves steadily from G1 to G5 potentially due to increasing data leakage, as looser partitions allow more overlap between train and test sets. In general, the models achieve slightly higher F1-score and accuracy for cutoff 2.

**Figure 6.**
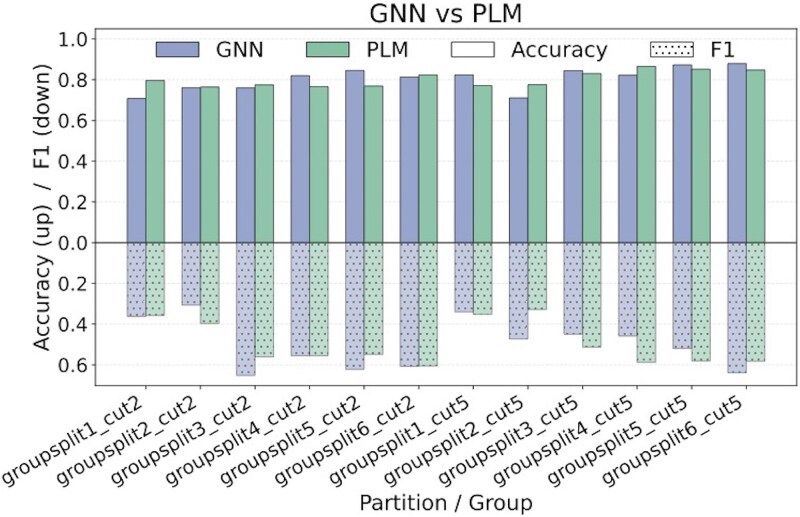
Comparison of the GNN and PLM performance across partitions using nested cross-validation: solid bars (positive axis) show accuracy and hatched bars (plotted downward) show F1-score.

Figure 6 compares GNN and PLM performance across partitions using nested cross-validation: solid bars (positive axis) show accuracy and hatched bars (plotted downward) show F1-score. In the C2 splits (e.g. G1_C2 and G2_C2) PLM achieves slightly higher accuracy (0.78 versus 0.71 in G1_C2) with comparable F1 values. From G3_C2 onward, GNN surpasses PLM on both accuracy and F1 (for instance, 0.82 versus 0.77 accuracy and 0.64 versus 0.55 F1 in G3_C2). At C5, PLM leads the early groups (G1_C5, G2_C5: 0.78 versus 0.72 accuracy; 0.47 versus 0.40 F1), while GNN is consistently stronger in later groups (e.g. G5_C5: 0.86 versus 0.84 accuracy; 0.65 versus 0.60 F1).

We note an important practical limitation: the large hyperparameter spaces of both model classes and the computational cost of nested cross-validation made exhaustive hyperparameter searches infeasible. As a result, we applied constrained tuning budgets for some splits, and it is possible that suboptimal hyperparameter choices partially explain cases where GNN’s accuracy or F1 are marginally lower than PLM’s. Despite this, the overall trend is that GNN generalizes better under mid-to-strict partitions (G3–G5), whereas PLM retains a modest advantage in the earliest splits.

**Figure 7.**
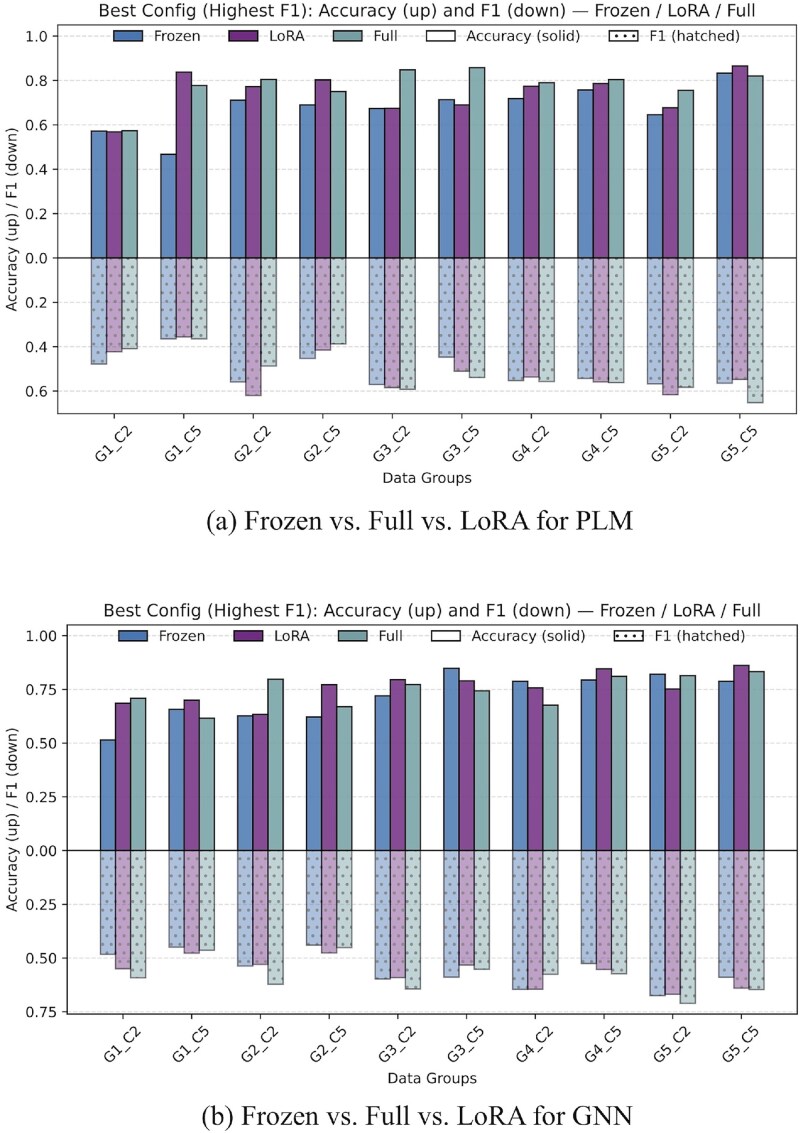
Comparison of frozen, full-parameter, and LoRA fine-tuning for (a) PLM and (b) GNN pipelines based on the highest F1-scores attained per group.

Figure 7 compares fine-tuning strategies across both PLM and GNN pipelines and reveals consistent, interpretable trends. For PLMs, both LoRA and full fine-tuning substantially outperform frozen representations, with the gap most pronounced on the stricter partitions (G1–G3) where frozen models show limited generalization. LoRA frequently approaches the performance of full fine-tuning, producing F1 scores in the $\sim$0.45–0.55 range on the most challenging splits, whereas frozen PLMs typically remain near $\sim$0.40–0.50; accuracy exhibits a similar separation.

In the GNN pipelines, the benefit of tuning is even clearer: tuned models (LoRA or full) achieve consistently higher accuracy (generally >0.75 across groups) and F1 scores on the order of $\sim$0.60–0.70, while frozen GNN variants underperform by $\sim$10–15 percentage points on these metrics. Together, these results indicate that parameter-efficient fine-tuning (LoRA) delivers a favorable compromise between performance and computational cost, closely matching full fine-tuning, and that incorporating structural context amplifies the gains obtained from tuning.

## Discussion

Accurate prediction of asparagine deamidation rates remains a critical challenge in protein therapeutic development. Chemical degradation such as deamidation can profoundly affect efficacy and stability of protein therapeutics, underscoring the need for robust, generalizable computational tools that reliably inform developability assessments early in the pipeline. Our work responds to several longstanding limitations and potential sources of bias in the deamidation prediction literature.

The introduction of a peptide grouping strategy for cross-validation effectively addresses sequence similarity-driven data leakage, a potentially overlooked problem that can distort model performance metrics and lead to overoptimistic benchmarking. By ensuring that sequences with similar peptides are maintained within the same validation fold, we provide a realistic assessments of model generalizability on novel sequences, a crucial requirement for application in diverse biotherapeutic portfolios. While this is a conservative approach to data partitioning and works well under the assumption that deamidation risk is dictated solely by the immediate sequence environment, it is important to recognize that local sequence context does not always fully describe the chemical or physical setting of a residue within the therapeutic protein. Influences from more distal regions of the molecule, which arise from the 3D structure or intermolecular interactions, can sometimes play a critical role in determining modification risk. Therefore, while sequence-based grouping is valuable for minimizing motif-driven leakage, additional structural information may be necessary to fully capture the complexity of deamidation.

Our benchmarking across four pipeline architectures highlights the advantages of integrating advanced PLMs and GNNs, especially for modeling the interplay of sequence and structure that drives degradation susceptibility. In a comprehensive evaluation of feature-based approaches, the Top1-structure model delivered the strongest overall performance. While this approach is computationally intensive—requiring structural prediction and subsequent feature calculation—it remains less resource-demanding than the Top25-augmented models, which utilize features from the Top25 predicted structures.

We next assessed model families (PLMs and GNNs) that offer scalability and richer structural expressiveness [23]. In particular, PLMs trained on large and diverse sequence datasets provide scalable, computationally efficient predictions, making them well suited for rapid screening of large candidate sets or applications where throughput is prioritized. Complementing PLMs, GNNs integrate local sequence context with explicit 3D spatial information, improving predictive accuracy, particularly when subtle structural effects are critical for determining modification risk. The ability of GNNs to capture structural features directly addresses cases where local sequence context alone is insufficient to predict degradation outcomes. Similar to feature-based models, GNNs require additional computation for structure prediction but are slightly less demanding because they do not require further feature engineering. Overall, GNNs perform on par with—and in some cases exceed—the Top1-structure models.

Taken together, PLMs are recommended for high-throughput, baseline assessments, whereas GNNs are preferable when maximizing predictive F1 score—balancing recall and precision—is the priority, such as in focused evaluation of final candidates or in cases where structural determinants play a critical role. GNNs also remain computationally advantageous relative to feature-based models: although they require structure prediction, they avoid the explicit feature-engineering/calculation step. Across all approaches, careful selection of window size and input features can yield substantial performance gains, strengthening the utility of these methods for diverse biotherapeutic applications.

The expansion of the training dataset in this work—to $\sim$591 asparagine sites across >100 protein therapeutics—also strengthens model performance. By including both antibody and non-antibody therapeutics under relevant stress conditions, the dataset supports broader applicability and more nuanced modeling across therapeutic classes and manufacturing environments.

Despite these advances, some limitations remain. Real-world validation in commercial candidate pipelines, and improvement of quantitative rate predictions rather than simple classification outputs, are important avenues for future work. While our peptide grouping strategy minimizes data leakage, our most accurate deamidation model (GNN) relies on protein structure predictions, which remain computationally intensive for screening thousands of sequences, especially for non-antibodies. Computational cost is expected to increase with conformational sampling techniques that capture dynamics and may further enhance therapeutic protein modification predictions. Among deamidation prediction literature, e.g. only very short conformational sampling or dynamics simulations have been used with antibody Fv structures [12]. There is an ongoing need for methods that can improve throughput of generating therapeutic protein structures and/or dynamics information for both antibodies and non-antibodies, which can be leveraged for predicting rare protein modification events that occur after complex conformational rearrangements or longer time-scales.

Overall, our findings suggest that the careful design of evaluation protocols, expansion of training data, and adoption of next-generation deep learning architectures can substantially improve the reliability and translational value of *in silico* deamidation prediction. The presented framework not only supports more informed risk assessments for therapeutic protein chemical stability but also provides a foundation for similar advances in modeling other complex degradation pathways and post-translational modifications.

## Conclusion

This work presents a systematic approach to predicting asparagine deamidation in protein therapeutics by combining curated datasets, strict evaluation protocols, and deep learning models. We applied a grouping strategy to control sequence-similarity leakage, enabling reliable performance assessment. The results show that PLMs provide strong sequence-based predictions, while GNNs that incorporate local structural context achieve $\sim$8% higher accuracy than PLM pipelines and consistent gains over motif-based baselines. Nested cross-validation confirms that these trends are stable across partitioning schemes.

The methodological framework introduced here, including leakage control, integration of PLM embeddings with structure-aware GNNs, and rigorous validation, can be applied to other therapeutic protein degradation pathways and post-translational modifications. This approach supports early stage *in-silico* liability assessment in biotherapeutic development by providing reproducible and interpretable predictions. Future work will extend the models to quantitative rate estimation, incorporate conformational dynamics, and evaluate performance on large-scale industrial pipelines.

**Key Points**

We developed an improved framework for deamidation prediction by curating a large unbiased dataset and preventing data leakage, showing that protein language models (PLMs) offer reliable, scalable assessment for protein therapeutics. To summarize our main contributions:

- **Rigorous Data Integrity Assessment:** We thoroughly investigated the influence of data splitting strategies on prediction validity, demonstrating that grouping sequence-similar peptides in the same fold is essential for preventing data leakage and yielding accurate generalization metrics. We also quantified how different sequence window sizes surrounding Asn sites affect model sensitivity and specificity, supporting rational parameter selection.
- **Comprehensive Model Pipeline Development:** We constructed and evaluated four major prediction pipelines, leveraging traditional sequence- and structure-based features to establish baseline performance and integrating state-of-the-art PLMs such as ESM2 with graph neural networks (GNNs). This framework enables learning of complex, context-dependent relationships directly from both sequence and structural attributes. Across these approaches, we systematically explored input feature sets, PLM variants, and GNN configurations to optimize predictive accuracy.
- **Practical Implications:** Our work establishes a robust computational framework capable of rapid, scalable, and reliable *in-silico* deamidation liability assessment. This significantly reduces the risk of late-stage pipeline failure due to chemical instability and enables its integration into early phase screening of biopharmaceutical candidates.

## Supplementary Material

Main_Paper_Deam_Briefings_in_Bioinformatics_bbag363

## Data Availability

The data underlying this article are not publicly available due to AstraZeneca’s proprietary policies. Subject to review and specific authorization from AstraZeneca, data access may be granted on a reasonable, case-by-case basis upon request to the corresponding author. The code is publicly available at https://github.com/AstraZeneca/Deam_GNN_briefings_bioinformatics. The repository includes the training scripts for the PLM baseline and the structure-aware GNN model, utility modules for data processing and model components, the data splitting script, example input files, example structure files, and the environment file required to reproduce the workflow.
