# Supplementary material for: Accurate prediction of asparagine deamidation in biologics using advanced machine learning models
Source: Brief Bioinform. 2026 Jul 7;27(4):bbag363. doi: 10.1093/bib/bbag363 (PMC13338906; doi:10.1093/bib/bbag363)
Supplement: Main_Paper_Deam_Briefings_in_Bioinformatics_bbag363 [file main_paper_deam_briefings_in_bioinformatics_bbag363.pdf]

Journal Title Here, 2025, pp. 1–1

doi: DOI HERE
Advance Access Publication Date: Day Month Year
Supplementary Material

SUPPLEMENTARY MATERIAL

Supplementary Material: Accurate Prediction of Asparagine Deamidation in Biologics Using Advanced Machine Learning Models

Shafayat Ahmed,[1,2**] Nicole Swope,[3**] Valentin Stanev,[1] Romina Hofele,[3] Dominique WuDunn,[3] Rohan Jain,[1] Jared Delmar[3,*] and Maryam Pouryahya[1,*]

[1]Data Science and Modelling, Biopharmaceutical Development, AstraZeneca, Gaithersburg, MD 20878, USA, [2]Department of Computer Science, Virginia Tech, Blacksburg, VA 24060, USA and [3]Analytical Sciences, Biopharmaceutical Development, AstraZeneca, Gaithersburg, MD 20878, USA

[*]maryam.pouryahya@astrazeneca.com, jared.delmar@astrazeneca.com

Abstract

The spontaneous deamidation of asparagine residues remains a major obstacle to the stability and efficacy of protein therapeutics. Currently available models in the literature for predicting deamidation liabilities can suffer from limited generalizability, likely due to biases such as sequence similarity within datasets. In this study, we built machine learning models using pretrained protein language models (e.g., ESM2) and graph neural networks (GNNs), trained on a comprehensive dataset of 591 asparagine sites from over 105 protein molecules. To address the critical issue of data leakage, we implemented a peptide grouping strategy yielding more realistic estimates of model performance for novel deamidation sites. Our analysis shows that, when sequence similarity bias is controlled, protein language models like ESM2 match traditional feature-based models that use amino acid composition, k-mers, PSSMs, and predicted secondary structure/solvent accessibility, while offering substantial computational advantages. Additionally, our GNN-based pipeline further increases prediction accuracy by up to 8% compared to language model-only tools and delivers a 15–25% improvement over motif-based approaches. This methodological framework enables more reliable and rapid in-silico prediction of deamidation liabilities, potentially reducing costly late-stage interventions in protein therapeutic development and and is broadly generalizable to the modeling of additional protein post-translational modifications.

© The Author 2025. Published by Oxford University Press. All rights reserved. For permissions, please e-mail: journals.permissions@oup.com

Table 1. Cross-validation performance (mean ± standard deviation) for GNN, PLM, Motif, and Top1-Structure models across different group–cutoff splits. Gx_Cy denotes the split configuration, where G specifies the grouping scheme used to prevent leakage and C specifies the diamidation rate cutoff used to separate good and bad molecules. For each split, the highest mean value within a metric column is shown in bold and underlined for clarity.

Data	Model	Accuracy	Precision	Recall	F1-Score	ROC-AUC	AUPRC
G1_C2	GNN	**.71** ± .18	**.53** ± .19	.82 ± .19	**.59** ± .09	**.77** ± .14	**.77** ± .14
	PLM	.57 ± .15	.34 ± .07	**.86** ± .12	.48 ± .06	.62 ± .14	.31 ± .08
	Motif-Based	.68 ± .24	.25 ± .28	.56 ± .52	.33 ± .34	.64 ± .17	.33 ± .19
	Top1-Structure	.74 ± .06	.44 ± .04	.73 ± .25	.53 ± .11	.74 ± .10	.48 ± .12
G2_C2	GNN	**.80** ± .09	**.57** ± .11	.71 ± .10	**.62** ± .08	.75 ± .12	**.55** ± .19
	PLM	.71 ± .18	.47 ± .17	**.74** ± .16	.56 ± .16	**.79** ± .10	.47 ± .15
	Motif-Based	.67 ± .18	.37 ± .30	.68 ± .37	.46 ± .33	.68 ± .21	.40 ± .24
	Top1-Structure	.72 ± .07	.38 ± .23	.48 ± .30	.41 ± .26	.63 ± .15	.40 ± .17
G3_C2	GNN	.77 ± .13	.56 ± .13	.80 ± .08	.64 ± .09	**.83** ± .11	.68 ± .15
	PLM	**.85** ± .08	**.67** ± .15	.58 ± .29	.59 ± .27	.72 ± .22	.63 ± .25
	Motif-Based	.67 ± .08	.37 ± .15	.65 ± .27	.47 ± .19	.66 ± .13	.34 ± .14
	Top1-Structure	.81 ± .09	.61 ± .13	**.82** ± .11	**.68** ± .08	.81 ± .06	**.76** ± .10
G4_C2	GNN	.79 ± .13	**.57** ± .18	**.78** ± .17	**.65** ± .16	**.76** ± .17	.57 ± .18
	PLM	**.79** ± .11	.55 ± .23	.63 ± .30	.56 ± .26	.73 ± .24	**.68** ± .24
	Motif-Based	.67 ± .14	.40 ± .09	.69 ± .26	.48 ± .14	.67 ± .12	.35 ± .08
	Top1-Structure	.78 ± .04	.53 ± .08	.72 ± .10	.60 ± .04	.76 ± .03	.58 ± .14
G5_C2	GNN	.81 ± .10	**.61** ± .17	**.89** ± .09	**.71** ± .14	**.86** ± .08	**.71** ± .18
	PLM	.76 ± .18	.54 ± .25	.64 ± .25	.58 ± .25	.77 ± .18	.68 ± .26
	Motif-Based	.68 ± .06	.40 ± .07	.71 ± .26	.49 ± .11	.69 ± .09	.36 ± .07
	Top1-Structure	**.84** ± .05	.54 ± .11	.58 ± .21	.55 ± .15	.74 ± .11	.60 ± .23
G1_C5	GNN	.70 ± .19	.38 ± .08	.76 ± .27	.48 ± .11	.69 ± .19	.39 ± .17
	PLM	.47 ± .13	.23 ± .08	**.93** ± .15	.36 ± .11	.61 ± .20	.32 ± .21
	Motif-Based	.65 ± .26	.17 ± .22	.57 ± .52	.25 ± .28	.63 ± .16	.25 ± .16
	Top1-Structure	**.80** ± .08	**.42** ± .20	.68 ± .31	**.49** ± .22	**.76** ± .14	**.47** ± .19
G2_C5	GNN	**.77** ± .09	.39 ± .13	.66 ± .29	**.48** ± .19	**.78** ± .10	**.48** ± .19
	PLM	.69 ± .24	**.41** ± .25	.63 ± .25	.45 ± .23	.70 ± .19	.38 ± .23
	Motif-Based	.63 ± .17	.27 ± .16	**.68** ± .38	.38 ± .22	.66 ± .18	.29 ± .08
	Top1-Structure	.76 ± .09	.36 ± .07	.40 ± .20	.35 ± .10	.62 ± .06	.35 ± .08
G3_C5	GNN	.85 ± .05	.56 ± .17	.66 ± .25	.59 ± .17	**.81** ± .13	.57 ± .22
	PLM	**.86** ± .06	**.67** ± .21	.49 ± .23	.54 ± .18	.75 ± .17	**.58** ± .22
	Motif-Based	.64 ± .09	.27 ± .13	.64 ± .30	.37 ± .17	.64 ± .13	.26 ± .11
	Top1-Structure	.79 ± .10	.49 ± .15	**.83** ± .09	**.60** ± .10	.80 ± .05	.52 ± .13
G4_C5	GNN	.81 ± .12	**.60** ± .23	.62 ± .18	**.57** ± .13	.77 ± .16	.52 ± .17
	PLM	.80 ± .07	.50 ± .09	**.70** ± .23	.56 ± .11	**.83** ± .10	**.62** ± .21
	Motif-Based	.63 ± .15	.30 ± .18	.63 ± .24	.40 ± .20	.63 ± .18	.29 ± .15
	Top1-Structure	**.83** ± .05	.54 ± .16	.50 ± .26	.49 ± .18	.70 ± .11	.59 ± .22
G5_C5	GNN	.83 ± .06	.55 ± .11	**.85** ± .18	**.65** ± .09	**.91** ± .06	**.74** ± .13
	PLM	.82 ± .18	**.71** ± .30	.69 ± .16	**.65** ± .23	.85 ± .13	.69 ± .23
	Motif-Based	.64 ± .11	.29 ± .17	.64 ± .31	.38 ± .20	.64 ± .18	.28 ± .11
	Top1-Structure	**.84** ± .05	.54 ± .11	.58 ± .21	.55 ± .15	.74 ± .11	.60 ± .23

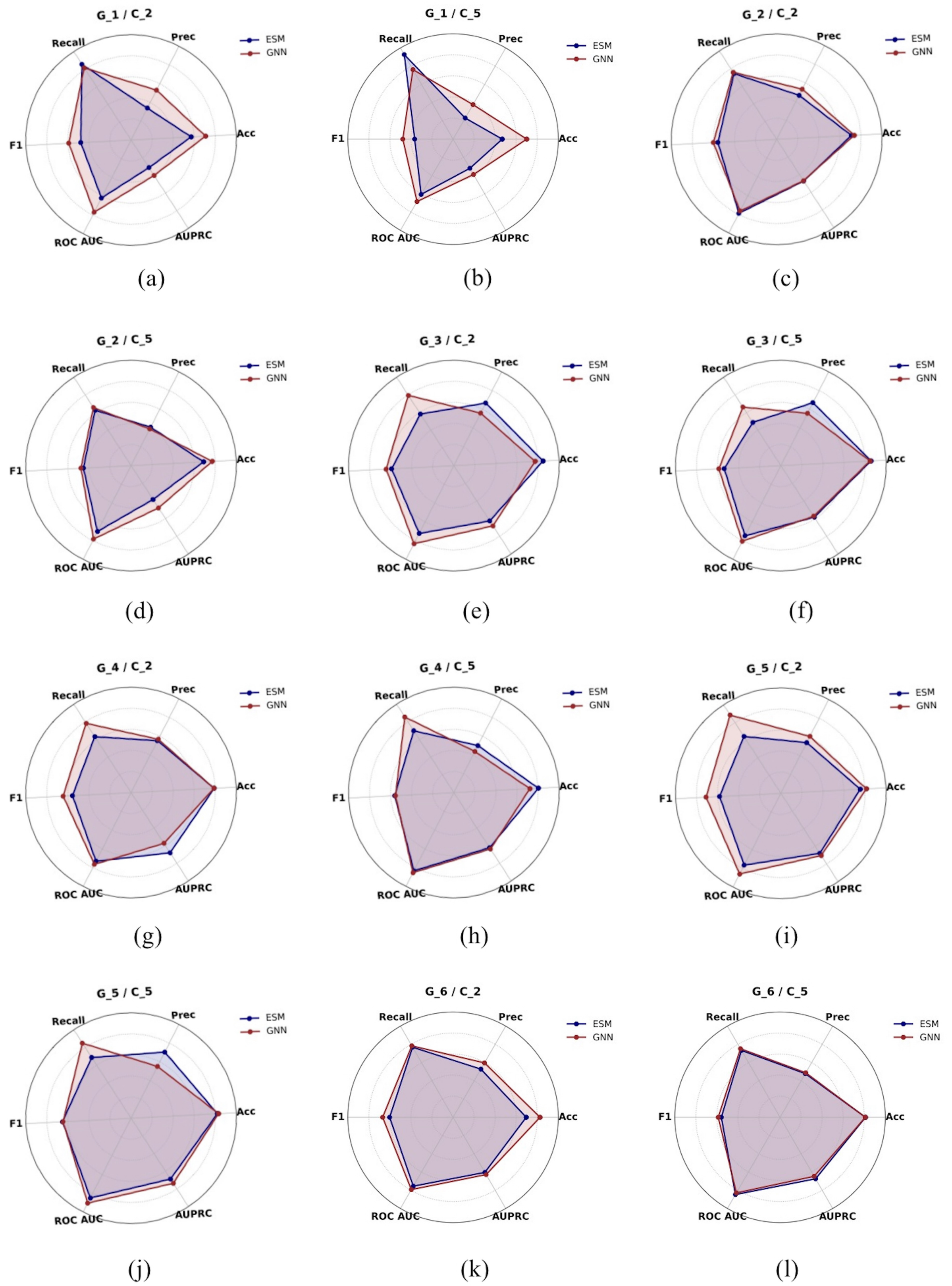

Fig. 1. Performance radar plots across grouping windows (G1–G6) and deamidation cutoffs (C2, C5) for multiple metrics.

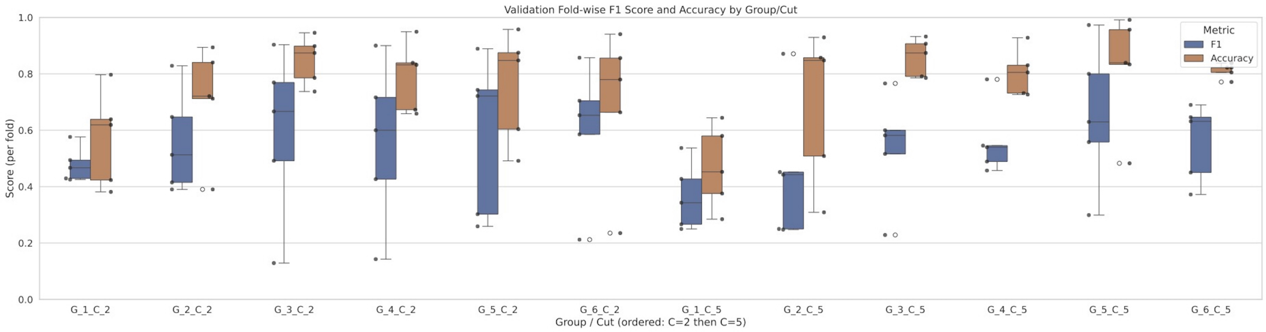

(a) PLM boxplot

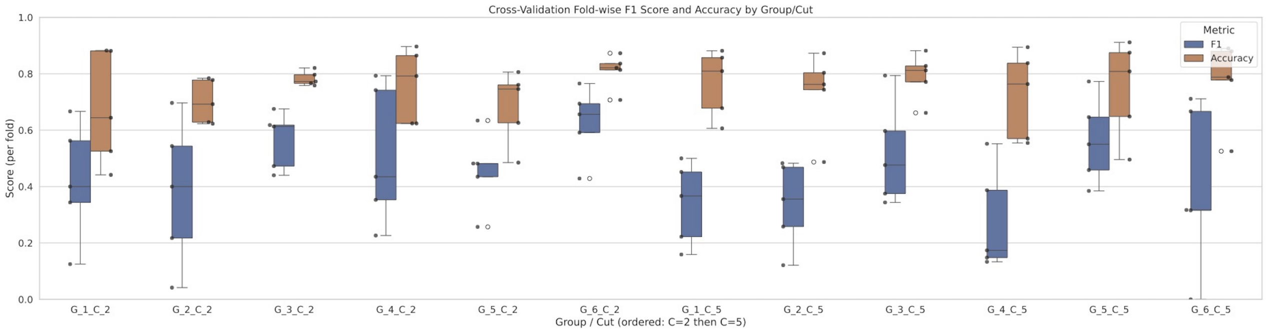

(b) GNN boxplot

Fig. 2. Cross-validation F1 and accuracy distributions for PLM and GNN across grouping strategies.
